# Transabdominal Intestinal Ultrasonography in Monitoring and Predicting Outcomes in Ulcerative Colitis—A Systematic Review

**DOI:** 10.3390/jcm15010035

**Published:** 2025-12-20

**Authors:** Sabrina Josefsen, Tobias Reinhold Larsen, Rune Wilkens, Jakob Benedict Seidelin, Johan Burisch, Mohamed Attauabi, Jacob Tveiten Bjerrum

**Affiliations:** 1 Department of Gastroenterology, Copenhagen University Hospital—Herlev Hospital, 2730 Herlev, Denmark; 2 Digestive Disease Center, Copenhagen University Hospital—Bispebjerg Hospital, 2400 Copenhagen, Denmark; 3 Department of Clinical Medicine, Faculty of Health and Medical Sciences, University of Copenhagen, 2100 Copenhagen, Denmark; 4 Copenhagen Center for Inflammatory Bowel Disease in Children, Adolescents, and Adults, Hvidovre Hospital, 2650 Hvidovre, Denmark; 5 Gastrounit, Medical Section, Copenhagen University Hospital—Hvidovre Hospital, 2650 Hvidovre, Denmark

**Keywords:** ulcerative colitis, intestinal bowel ultrasonography, predict

## Abstract

**Background/Objectives:** Intestinal ultrasound (IUS) is increasingly used to monitor ulcerative colitis (UC), but its predictive value remains unclear. This systematic review evaluated the ability of IUS parameters and scores to predict short- and long-term treatment response, remission, and adverse outcomes in hospitalized and outpatient UC populations. **Methods:** A systematic review was conducted according to Cochrane and PRISMA guidelines. MEDLINE and Embase were searched for prospective studies assessing IUS as a predictor of clinical or endoscopic response, remission, relapse, or adverse outcomes in adult UC. Two reviewers independently performed screening, data extraction, and QUADAS-2 assessment. **Results:** Eighteen prospective studies were included: eleven outpatient studies and seven involving hospitalized patients treated with intravenous corticosteroids (IVCS). In hospitalized patients, bowel wall thickness (BWT) was the most consistent predictor of treatment failure, rescue therapy, colectomy, and clinical response. Baseline BWT showed variable performance, but once IVCS was initiated, early BWT change within 48–72 h was the strongest marker of disease trajectory. Non-responders had higher BWT and smaller reductions. A BWT ≥ 4 mm, absolute reduction ≤ 1 mm, or relative reduction ≤ 20% at 48 h reliably identified patients needing rescue therapy (area under the curve (AUC) values of 0.77 (95% confidence interval (CI) 0.71–0.74), 0.71 (95% CI 0.56–0.86), and 0.74 (95% CI 0.60–0.88)). Colectomy risk was similarly predicted: BWT < 3 mm at 48 h was associated with no colectomies, whereas BWT ≥ 4 mm or persistently elevated BWT at day 6 markedly increased risk (Odds ratio (OR) 9.5-fold (95% CI 1.4–64.0) and OR 8.3 (95% CI 1.7–40.0), respectively). Other sonographic features (loss of haustration, increased vascularity) added supplementary but less consistent value. In outpatients, BWT also demonstrated the strongest predictive accuracy. BWT ≤ 3.6 mm at 2 weeks and <3.0 mm at 6 weeks were associated with early endoscopic remission (area under the receiver operating characteristic (AUROC) of 0.87 (95% CI 0.71–1.00) and 0.82 (95% CI 0.63–1.00), respectively). Dynamic changes with ≥23–25% relative reduction predicted clinical or endoscopic response (AUROC of 0.81 (95% CI 0.61–1.00) and OR of 13.9 (95% CI 1.13–1986.85), respectively). Persistent BWT > 3.5 mm or minimal reduction (<20% or <1 mm) indicated a low likelihood of long-term remission. Composite vascularity-based indices, particularly the Milan Ultrasound Criteria (MUC), strengthened prediction: MUC ≤ 4.3 or ≥2-point reduction at 12 weeks predicted long-term remission (AUROC 0.88 (95% CI 0.750–0.952) and 0.82 (95% CI 0.68–0.91), respectively), while MUC ≥ 7.7 indicated high risk of treatment failure or colectomy (AUROC 0.77 (95% CI: 0.73–0.82)). **Conclusions:** Across clinical settings, BWT consistently emerged as the strongest IUS predictor of UC treatment outcomes. Early BWT change within 48–72 h in hospitalized patients and absolute BWT values at 2–6 weeks in outpatients showed high predictive accuracy for response, remission, and colectomy. Composite indices incorporating vascularity further improved prediction. These findings support the incorporation of IUS into early treatment-response algorithms and underscore the need for standardized cut-offs and multicenter validation.

## 1. Introduction

Ulcerative colitis (UC) is a chronic inflammatory bowel disease (IBD) characterized by continuous colonic inflammation, beginning in the rectum and extending orally to a variable extent [1]. Symptoms of active disease include bloody diarrhea, abdominal pain, and urgency [2]. The global prevalence of UC is rising, with an estimated 5 million individuals currently affected [1,3].

UC demonstrates considerable inter-patient and intra-patient heterogeneity in its clinical course, ranging from prolonged periods of remission with no symptoms and limited need for surveillance to recurring exacerbations or continuous inflammation with substantial morbidity [4]. Early identification of disease activity and optimization of therapy in these later cases are necessary to reduce the risk of complications such as hospitalization, colectomy, and impaired quality of life [5,6]. Consequently, optimal strategies for monitoring the disease and treatment response are central for improving long-term outcomes.

Currently, disease activity is assessed through a combination of clinical scores and biochemical markers, including serum C-reactive protein (CRP) and stool biomarkers such as fecal calprotectin (FC). These non- or semi-invasive measures provide indirect information about underlying inflammation, but lack sufficient specificity and sensitivity [7,8]. Colonoscopy thus remains the gold standard, allowing direct visualization of the mucosa to assess endoscopic disease activity and obtain biopsies for histological evaluation [4,9]. Endoscopic and histological remission are associated with improved long-term outcomes and reduced risk of relapse, and consequently are established as vital treatment targets [4,9]. Despite colonoscopy’s central role, it has significant limitations, including the need for bowel preparation, procedural delays, invasiveness, and patient discomfort [10]. In contrast, intestinal ultrasonography (IUS) is a non-invasive, well-tolerated, real-time imaging modality that requires no preparation and can be performed during routine outpatient visits [9,11,12]. This has led to an increased interest in IUS as an alternative monitoring strategy, and recent evidence demonstrates that IUS correlates well with endoscopic assessment of disease activity [9,13,14].

IUS can assess several sonographic features that are indicative of inflammation in the intestines; these include bowel wall thickness (BWT), bowel wall vascularization assessed by color Doppler signal (CDS), bowel wall stratification (BWS), presence of dehaustration, mesenteric lymphadenopathy, and inflammatory fat (I-fat) [9,12]. In adults, ultrasonographic remission is typically defined by the normalization of bowel wall thickness (<3 mm) together with an absence of color Doppler signal (CDS = 0), reflecting the resolution of active mural inflammation [4]. To standardize IUS assessment and improve the sensitivity and specificity of disease evaluation, several scoring systems have been developed. The Milan Ultrasound criteria (MUC) combine BWT and CDS to provide a quantitative measure of disease activity [15], and the International Bowel Ultrasound Segmental activity score (IBUS-SAS) integrates BWT, CDS, BWS, and I-fat [16], while the UC-IUS includes BWT, CDS, haustration, and I-fat [17]. Studies have shown these IUS scoring systems and parameters not only correlate well with endoscopy, but also with biochemical and clinical UC activity markers, thus validating the clinical relevance of IUS [14].

In treat-to-target strategies, only measures with proven prognostic value are meaningful treatment targets, whereas unvalidated markers—such as histologic or molecular remission—remain debated precisely because their long-term predictive impact is uncertain. Consequently, identifying tools that reliably forecast treatment response, remission, or relapse is essential, as such measures enable proactive, personalized disease management and more effective long-term outcomes in UC [4]. IUS has emerged as a promising tool in this context because it allows for repeated, real-time, and non-invasive assessment of intestinal inflammation directly at the point of care [18]. Although the monitoring capabilities of IUS are well established, its predictive power—that is, the ability of IUS findings to estimate the risk of future disease activity, treatment response, or relapse—remains less clearly defined, but is critical for enhancing precision medicine approaches in UC.

The objective of this systematic review was to synthesize and critically appraise the existing evidence on the predictive value of IUS in UC, with particular emphasis on its ability to forecast short- and long-term treatment response, remission, and relapse, as well as adverse disease course.

## 2. Methods

This systematic review and network meta-analysis was conducted according to the Cochrane recommendations [19], and is reported according to Preferred Reporting Items for Systematic Reviews and Meta-Analyses statement [20,21] (Supplementary Table S7). The study protocol was defined and registered prior to study initiation at the PROSPERO database (registration number 1082752).

### 2.1. Literature Search and Strategy

Existing literature was systematically searched in Medline (via PubMed) and Embase. A comprehensive research strategy using MeSH and free text terms related to UC, IUS, and clinical outcomes (remission/response/relapse) were developed (see Supplementary Table S1).

At least two authors (SJ, TL, or MA) systematically and independently screened citations by title and abstract and subsequently in full text, with manual screening for and exclusion of duplicates.

### 2.2. Inclusion- and Exclusion Criteria

Studies were eligible for inclusion if they enrolled adult patients (≥18 years) with a confirmed diagnosis of UC according to recognized criteria [1]. Eligible studies were required to include baseline IUS prior to or in the early phase of medical therapy. Accepted therapies included corticosteroids, 5-aminosalicylic acid (5-ASA), immunomodulators, biological therapies, or small molecules. Disease response/remission/relapse following medical intervention could be evaluated using clinical indices (e.g., partial Mayo score, Simple Clinical Colitis Activity Index [SCCAI], Short Inflammatory Bowel Disease Questionnaire [SIBDQ]), endoscopic scores (e.g., Mayo endoscopic score [MES] and UC Endoscopic Index of Severity [UCEIS]), biochemical markers (e.g., CRP and FC), or IUS parameters and validated scoring systems (e.g., BWT, CDS, MUC, and IBUS-SAS). Data comparing the predictive capability of different evaluation methods was considered, although not required for study inclusion.

Only prospective study designs were considered, including prospective cohort studies and nested analyses within randomized controlled trials, whereas retrospective studies, case reports, narrative reviews, editorials, and conference abstracts without full text were excluded. Populations with Crohn’s disease, indeterminate colitis, or immune-mediated colitis were excluded. Abstracts without full texts were included if sufficient data and information were provided in the abstract.

### 2.3. Pre-Defined Outcomes and Definitions

The predefined primary outcomes were treatment failure, clinical and endoscopic response, remission, and relapse, defined according to EMS, MES, and UCEIS, and clinically validated symptom- or biomarker-based criteria as reported in each study. Secondary outcomes included a need for rescue therapy, treatment escalation, hospitalization, and colectomy. Definitions of these outcomes are provided for each study in Supplementary Tables S5 and S6.

IUS predictors included BWT, CDS, BWS, I-fat, dehaustration, and validated composite scores (MUC, UC-IUS, and IBUS-SAS). Cut-off values for these parameters were recorded as reported.

### 2.4. Data Extraction

A standardized, predefined form was used by two independent authors (SJ and TL) to extract data from the included studies. Disagreements were resolved by discussion among the two reviewers, and in some cases by adjudication by a third reviewer.

### 2.5. Risk of Bias Assessment

The risk of bias was assessed independently by two reviewers. For studies evaluating the predictive performance of IUS with respect to treatment response, remission, or relapse, Quality Assessment of Diagnostic Accuracy Studies-2 (QUADAS-2) was applied with the following domains: patient selection, index test (IUS), reference standard, and flow/timing. Signaling questions adapted to the review question were pre-specified (Supplementary Table S2. Each domain was rated as low, high, or unclear risk of bias. Disagreements were resolved by discussion among the two reviewers, and in some cases, by adjudication by a third reviewer (Supplementary Table S3).

## 3. Results

### 3.1. Study Inclusion

**Figure 1:**
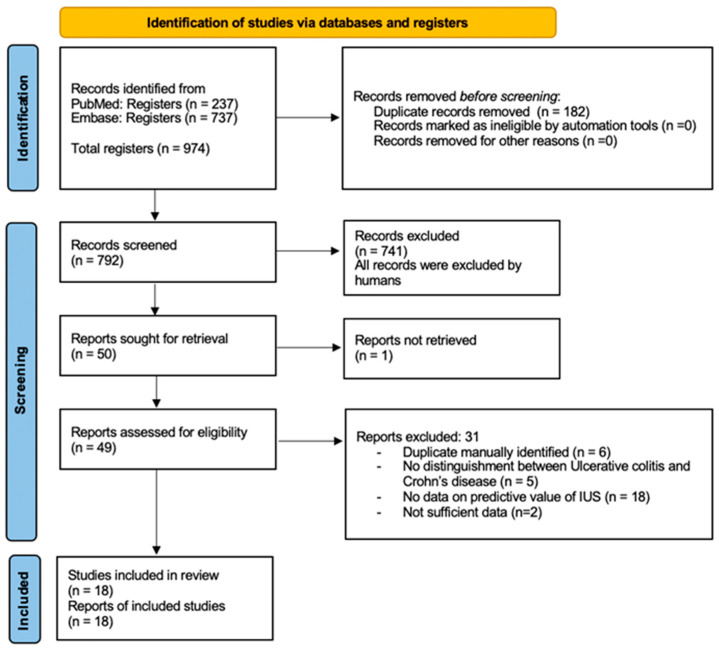
PRISMA 2020 flow diagram for new systematic reviews, which included searches of databases and registers only [1]. IUS, intestinal ultrasonography.

The database search identified 974 records. After the removal of duplicates and screening of titles and abstracts, 49 studies underwent full-text review. Eighteen studies met the predefined inclusion criteria and were included in the qualitative synthesis (10 full-text publications and eight conference abstracts/posters). The study selection process is summarized in Figure 1.

A substantial number of studies were excluded because, although they involved intestinal ultrasound, they evaluated only the concurrent accuracy of IUS—examining cross-sectional correlations with endoscopic, biochemical, or clinical markers—and did not assess the ability of IUS to predict future treatment response, remission, or relapse. Because the objective of this review was prognostic rather than diagnostic, such studies were not eligible for inclusion.

### 3.2. Bias

The methodological quality of the included studies, assessed using the QUADAS-2 tool, is summarized in Supplementary Table S3. Overall, many studies lacked sufficient methodological detail, resulting in an “unclear” risk-of-bias rating in several domains. Most studies adequately described the conduct and interpretation of the index test (IUS) and reference standards, yielding generally low concern for bias in these areas. In contrast, the patient selection domain frequently lacked complete reporting of sampling approaches, exclusion criteria, and whether consecutive or random enrolment was used, leading to unclear risk assessments. Blinding procedures were rarely reported. In many studies, it remained uncertain whether IUS examiners were blinded to clinical or endoscopic outcomes, raising the possibility of bias in test interpretation. Despite these concerns, applicability issues were minimal, as the patient populations, interventions, and outcomes generally aligned well with the aims of this review.

### 3.3. Study Characteristics

Supplementary Table S4 summarizes baseline characteristics of the included studies.

A total of seven studies examined patients with acute severe UC (ASUC) requiring hospitalization and treatment with intravenous corticosteroids (IVCS) [22,23,24,25,26,27,28]. Follow-up time was typically <1 week, except for two studies [24,28], which also included follow-up months after baseline (Supplementary Tables S4 and S5).

A total of 11 studies examined outpatients receiving or initiating oral corticosteroids, biologics, thiopurines, 5-ASA, Janus kinase (JAK) inhibitors, or combinations of these [29,30,31,32,33,34,35,36,37,38,39]. Follow-up time ranged from 8 weeks to 3 years. Three studies did not provide information about disease extent [33,35,38]. Eight studies provided information on disease extent [29,30,31,32,34,36,37,39]; three included patients with isolated proctitis [34,36,37] (Supplementary Tables S4 and S6).

Reported outcomes included a need for rescue therapy [22,23,25,26,39], endoscopic remission [24,29,30,31] or response [29,30,31,36,38], clinical remission [32,39] or response [25,33,35], treatment failure [24,27,28,29,34], and colectomy [22,24,28,37,39]. No study assessed the ability of IUS to predict relapse. Definitions of these outcomes varied considerably across studies, limiting direct comparability. Because of this heterogeneity—in outcome definitions, IUS parameters, assessment timing, and reporting approaches—a quantitative meta-analysis could not be performed. Instead, the results are synthesized descriptively and grouped into two main clinical settings: hospitalized UC patients receiving IVCS, and outpatients initiating or continuing other medical therapies.

### 3.4. Hospitalized UC Patients

Across all seven studies evaluating hospitalized patients with ASUC, BWT consistently emerged as the most reliable IUS parameter for predicting clinically meaningful outcomes, compared to BWS, I-fat, and CDS [22,23,24,25,26,27,28]. These outcomes included a need for rescue therapy (typically infliximab or cyclosporine) [22,23,25,26], colectomy [22,24,28], clinical response [25], treatment failure [24,27,28], and endoscopic response or remission [24]. Only one study assessed the UC-IUS index in this context, demonstrating its ability to predict the need for rescue therapy with an area under the receiver operating characteristic curve (AUROC) of 0.84 (95% confidence interval (CI) 0.74–0.94) [22].

#### 3.4.1. Need for Rescue Therapy

The predictive role of BWT varied depending on whether it was measured before or after initiation of IVCS. One study showed baseline BWT prior to IVCS was a strong predictor: Smith et al. observed a significantly higher mean and sigmoid BWT within 24 h of hospital admission in patients who ultimately required infliximab (4.6 vs. 6.2 mm, *p* < 0.001; 5.0 vs. 7.0 mm, *p* = 0.033) [23]. Notably, these measurements were taken within 24 h of admission and therefore potentially after the initiation of IVCS, which may partially account for their strong predictive performance. By contrast, other studies found that baseline BWT prior to IVCS administration did not distinguish responders from non-responders [25,26]. This inconsistency underscores the importance of when “baseline” imaging is performed in relation to IVCS initiation.

Despite discordant baseline findings, a consistent pattern emerged once IVCS was initiated: early BWT reductions within the first 48–72 h were among the strongest predictors of subsequent rescue therapy. For example, one study reported AUROC values of 0.87 (95% CI 0.79–0.96) for sigmoid colon and 0.84 (95% CI 0.74–0.94) for sum of BWT for all colon segments, in predicting subsequent rescue therapy [22]. Likewise, Ilvemark et al. identified significantly higher BWT in non-responders 48 ± 24 h after treatment initiation (4.4 mm vs. 3.1 mm, *p* = 0.002) [25], while An et al. reported meaningful early differences by day 3 [26].

Using BWT at 48 ± 24 h, an absolute BWT ≥ 4 mm, an absolute reduction ≤ 1 mm, and a relative reduction ≤ 20%, each demonstrated balanced predictive accuracy, with AUC values of 0.77 (95% CI 0.71–0.74), 0.71 (95% CI 0.56–0.86), and 0.74 (95% CI 0.60–0.88), respectively [25]. Likewise, absolute reductions < 1.4 mm and relative reductions < 20% from admission to day 3 yielded AUROC values of 0.76 and 0.78 [26].

Although studied far less frequently, additional IUS features offered some predictive value. Loss of haustration at baseline was more prevalent in non-responders requiring rescue infliximab (*p* = 0.66) [25]. Early differences in CDS and haustration patterns were observed between responders and non-responders 48 ± 24 h after IVCS initiation, though these differences were no longer apparent by day 6 [25].

#### 3.4.2. Risk of Colectomy

BWT was also the most consistent predictor of short- and long-term colectomy risk. At the time of admission, baseline BWT ≥ 7.0 mm was associated with a higher likelihood of colectomy within 60 days (log-rank *p* = 0.075) [28]. After IVCS therapy initiation, the predictive ability of BWT was strengthened even further. After 3 days of treatment, both sigmoid BWT and sum of BWT, for all colon segments, showed excellent accuracy for identifying patients at a high risk of colectomy according to the Lindgren score, with AUROC values of 0.86 (95% CI 0.75–0.96) and 0.88 (95% CI 0.80–0.97), respectively [22]. Although the UC-IUS index after 3 days of IVCS also predicted colectomy (AUROC of 0.85, 95% CI 0.76–0.94), BWT alone performed slightly better in this context [22].

Assessments at 48 ± 24 h provided particularly clear separation between those who would and would not require colectomy. A sigmoid BWT < 3.0 mm at 48 ± 24 h was associated with zero colectomies within one year, whereas a BWT ≥ 4.0 mm had a dramatically increased risk (9.5-fold; 95% CI 1.4–64) [24]. Persistently elevated BWT at day 6 (≥3.5 mm) also predicted colectomy, OR 8.3, (95% CI 1.7–40) [24].

#### 3.4.3. Clinical Response

Only one study evaluated clinical response prediction using IUS. Baseline IUS features were non-discriminatory, but by 48 ± 24 h, BWT had strong predictive value. Absolute BWT ≥ 4.0 mm at this timepoint achieved an AUROC of 0.85 (95% CI 0.76–0.95), with absolute reduction of ≤1.0 mm and relative reductions of ≤20% showing similar performance (AUROC of 0.81 (95% CI 0.69–0.93) and 0.85 (95% CI 0.74–0.95)) [25]. At this timepoint, other IUS parameters were observed to be differences between responders and non-responders—including normal bowel wall stratification, absence of I-fat, and presence of haustrations (*p* = 0.01, *p* = 0.003, and *p* < 0.01, respectively) [25]. A day 6, differences in BWS and i-fat between responders and non-responders were no longer significant, but differences in haustration pattern remained significant (*p* < 0.0001) [25].

#### 3.4.4. Long-Term Treatment Failure

Definitions of treatment failure varied, encompassing combinations of rescue infliximab, second-line therapy initiation, corticosteroid escalation, biologic switching, urgent colectomy, or hospitalization. Nonetheless, the findings were consistent: higher baseline BWT and CDS predicted long-term treatment failure. Baseline BWT ≥ 7.0 mm predicted progression to second-line therapy (*p* = 0.021) [28], while BWT measured at 48 h (6.3 vs. 2.4 mm, *p* = 0.0001) also strongly predicted failure [27]. Persistently elevated BWT at 48 ± 24 h, 6 days, and 3 months was associated with 3.5 to 3.9-fold increased risk of treatment failure [24].

#### 3.4.5. Endoscopic Remission

Only one study evaluated IUS prediction of endoscopic remission in hospitalized patients. BWT normalization (<3 mm) at 3 months strongly predicted endoscopic remission at the same timepoint, OR 12.5 (95% CI 2.3–67) [24].

### 3.5. Outpatients

Among outpatients initiating or modifying medical therapy, BWT again emerged as the most robust predictor of future outcomes, including treatment response, clinical and endoscopic remission, and colectomy. However, compared with hospitalized cohorts, a broader range of IUS parameters—particularly vascularity and composite indices—demonstrated complementary prognostic value.

#### 3.5.1. Endoscopic Remission

Across outpatient studies, a clear pattern emerged in which early improvements on IUS reliably preceded mucosal healing. Absolute BWT values at follow-up were particularly informative. Two weeks after therapy initiation, a descending colon BWT ≤ 3.6 mm predicted endoscopic remission at 8–26 weeks, AUROC 0.87 (95% CI 0.71–1.00, *p* = 0.006) [30]. By week 6, sigmoid and descending BWT thresholds ≤ 3.0 and ≤3.2 mm predicted remission with AUROC values of 0.82 (95% CI 0.63–1.00) and 0.89 (95% CI 0.74–1.00), respectively, and at 8–26 weeks, even stricter cut-offs of ≤2.6 and ≤2.7 mm achieved excellent accuracy (AUROC 0.95, 95% CI 0.88–1.00, and 0.96, 95% CI 0.87–1.00, respectively) [30]. Additionally, a >28% reduction in BWT after the biological therapy induction period predicted endoscopic response (AUROC 0.74, 95% CI 0.589–0.896) [38].

Beyond total BWT, deeper structural changes also proved meaningful: each millimeter increase in submucosal thickness at week 6 strongly predicted endoscopic remission (OR 0.09, 95% CI 0.01–0.65) [30]. Supporting the importance of submucosal features provides important insights into submucosal hyperechogenicity, suggestive of chronicity or fibrosis, and predicted failure to achieve endoscopic remission (OR 0.10, 95% CI: 0.01–0.87) [29].

Composite indices integrating vascularity refined prediction further. A MUC ≤ 6.2 at 12 weeks was associated with substantial odds of remission (OR 10.4, 95% CI 1.09–99.29) [31]. Similarly, a ≥2-point MUC reduction predicted remission at 12 weeks (AUROC 0.82, 95% CI 0.68–0.91), with MUC ≤ 4.3 identified as the optimal cut-off (AUROC 0.88, 95% CI 0.750–0.952) [31].

#### 3.5.2. Endoscopic Response/Improvement

A similar narrative was applied to endoscopic response. Sigmoid BWT ≤ 3.5 mm at 8–26 weeks demonstrated near-perfect accuracy for predicting endoscopic response (AUROC 0.96, 95% CI 0.88–1.00) [30]. Relative reductions ≥23% were also highly predictive (AUROC 0.81, 95% CI, 0.61–1.00) [30]. As with remission, a decrease in submucosal thickness at 6 weeks was strongly associated with endoscopic improvement (OR 0.14, 95% CI 0.03–0.75) [30].

Changes in vascularity showed a supportive, though slightly less robust value: a reduction in CDS after six weeks predicted endoscopic improvement (OR 0.35, 95% CI 0.14–0.88) per category increase) [30]. Echogenicity parameters again offered prognostic refinement. Submucosal hyperechogenicity predicted lack of improvement (OR 0.16, 95% CI 0.14–0.88) [29], while relative submucosal echogenicity predicted endoscopic response with an AUROC of 0.76 (95% CI 0.61–0.92), using >108 greyscale units as the optimal cut-off (OR 0.07, 95% CI 0.01–0.45) [36].

#### 3.5.3. Risk of Colectomy

Two outpatient studies evaluated the ability of IUS to predict colectomy risk, and their findings aligned closely with the broader patterns observed across this review. In the first, baseline BWT predicted colectomy over a median 1.8-year follow-up with good accuracy (AUROC 0.80, 95% CI 0.71–0.90), with ≥4.6 mm emerging as the optimal threshold. Incorporating vascularity offered a modest incremental advantage: baseline MUC predicted colectomy with a similar AUROC (0.83, 95% CI: 0.75–0.92), and a cut-off ≥ 7.7 best identified high-risk patients, whereas CDS alone showed weaker discriminatory performance (AUROC 0.77, 95% CI: 0.73–0.82) [37].

A second study further reinforced the prognostic relevance of early IUS abnormalities. Here, BWT measured at the time of UC diagnosis independently predicted colectomy within three months (OR 2.0, 95% CI: 1.2–3.3), with >6 mm identified as the optimal cut-off (AUC 0.85). Additional IUS features—including CDS, loss of BWS, and presence of I-fat—were also predictive, with ORs of 4.9 (95% CI: 2.0–2.6), 3.4 (95% CI: 1.8–7.9), and 3.6 (95% CI: 1.7–9.5), respectively. Notably, the IBUS-SAS outperformed BWT alone, achieving an AUC of 0.88, suggesting that incorporating parameters reflecting inflammatory changes beyond the mucosa enhances predictive precision. An IBUS-SAS score > 42 was identified as the optimal threshold for predicting colectomy within three months of diagnosis [39].

#### 3.5.4. Treatment Failure

IUS parameters also predicted broader markers of treatment failure. A baseline MUC > 6.2 was associated with a negative disease course over a median 1.6-year follow-up (HR 3.87, 95% CI: 2.25–6.64) [31]. Structural abnormalities were equally significant: the presence of submucosal hyperechogenicity prior to treatment was associated with increased risk of failure during the following 8–26 weeks (OR 4.4, 95% CI: 1.08–18.32 for failure of one biologic; OR 5.6, 95% CI: 1.54–20.52 for failure of >1 biologic) [40].

Furthermore, achieving transmural remission at three months after UC onset substantially reduced the need for IVCS treatment during the first year (6% vs. 19%, *p* = 0.04) [39].

#### 3.5.5. Clinical Response and Remission

Ultrasound markers also correlated with clinical outcomes. Contrast-enhanced ultrasound showed that responders to vedolizumab exhibited greater perfusion changes after 14 weeks (*p* = 0.037) [35]. A ≥25% BWT reduction after six weeks of vedolizumab strongly predicted clinical response (OR 13.9, 95% CI 1.13–1986.85) [34]. Lower baseline BWT predicted clinical remission at three months (OR 0.19, 95% CI 0.05–0.72), and baseline MUC similarly predicted clinical remission (OR 0.48, 95% CI 0.21–0.83) [32]. Achieving transmural remission at three months also translated into higher rates of steroid-free clinical remission at 6, 9, and 12 months [39].

## 4. Discussion

### 4.1. Summary of Findings

Across the 18 included studies, IUS consistently demonstrated promise as a noninvasive modality for predicting both short- and long-term outcomes in UC. A central finding across cohorts was the pivotal role of BWT: in hospitalized patients receiving IVCS, early reductions in BWT within 48–72 h reliably predicted clinical response [25], need for rescue therapy [25,26], and colectomy [24,27], whereas minimal or absent early change signaled a poor trajectory. Among outpatients initiating medical therapy, both absolute BWT values and relative reductions over time were strongly associated with endoscopic and clinical remission, typically with thresholds of 2.7–3.5 mm or ≥20% reduction [30,31,32]. Although BWT emerged as the most reproducible and reliable parameter across studies, other sonographic features—particularly bowel wall vascularity, echogenicity, and composite indices such as the MUC and IBUS-SAS—also demonstrated predictive values [31,37,39]. Collectively, these findings position BWT as the cornerstone of predictive IUS assessment, while highlighting the complementary contribution of additional parameters.

### 4.2. Timing and Dynamics of BWT Change

Building on this central role of BWT, one of the most compelling themes emerging from the evidence concerns the timing of BWT change—particularly in hospitalized patients with ASUC. Multiple studies converged on the same conclusion: the first 48 h following IVCS initiation constitute the critical prognostic window. Ilvemark et al. demonstrated that patients who exhibited little or no reduction in BWT within this brief period failed to show further meaningful improvement over the subsequent six days, whereas early responders continued to improve [24]. This indicates that dynamic, early IUS changes—rather than later measurements—are the primary determinants of overall BWT evolution and the most informative reflections of treatment response.

This temporal insight also helps explain discrepancies across studies evaluating baseline BWT. Investigations measuring BWT immediately upon hospital admission, prior to corticosteroid exposure, often found baseline BWT non-predictive of rescue therapy needs [22,25,26]. In contrast, Smith et al. measured BWT within 24 h of admission—likely after the first corticosteroid doses—and observed significant baseline differences between responders and non-responders [23]. Similarly, Zacharopoulou et al., who assessed BWT within 48 h of IVCS initiation, reported higher BWT in patients who ultimately failed therapy [27]. Thus, much of the apparent inconsistency in baseline findings is clarified when considering the timing of IUS relative to treatment.

### 4.3. Beyond the Mucosa: A Broader View of UC Pathology

These observations naturally raise a deeper question: what aspects of bowel pathology does BWT—and its early change—actually capture? Traditionally, UC is described as a purely mucosal disease, yet emerging IUS and histopathological research challenge this long-standing view. De Voogd et al. demonstrated that submucosal thickening is predictive of endoscopic response, and colectomy specimens reveal that the submucosa is the most affected mural layer [30]. Adding to this, Komatsu et al. developed the Submucosal Index—the ratio of submucosal thickness to total BWT—and found that this index had superior specificity compared with both MUC and BWT in predicting endoscopic remission [41]. Pruijit et al. added another dimension by showing that relative submucosal echogenicity (RSE) at baseline—*before* any treatment is given—can already distinguish future responders from non-responders [36]. This suggests that echogenicity-based markers may capture structural or inflammatory alterations that precede measurable changes in wall thickness. If confirmed, such parameters could enable clinicians to anticipate treatment trajectories earlier, potentially guiding therapeutic decisions without waiting for the 48–72 h post-treatment BWT reassessment that current algorithms rely on. These findings collectively suggest that UC pathophysiology extends beyond the mucosa and that deeper mural involvement may carry important prognostic significance.

### 4.4. Evolving Concepts of Remission

If UC involves deeper mural layers, the concept of remission must evolve accordingly. STRIDE-II continues to prioritize endoscopic healing as the principal long-term treatment target, yet also recognizes that histological remission provides additional prognostic insight—being associated with lower risks of relapse, corticosteroid exposure, and hospitalization [4]. Because histological scoring systems lack standardization and its independent prognostic contribution remains uncertain, STRIDE-II currently designates it as an adjunct rather than a formal therapeutic target [4].

Alongside histological healing, emerging evidence suggests that transmural remission may offer an even more comprehensive reflection of true disease resolution [37]. This shift has important implications for IUS, which uniquely visualizes mural and extra-mucosal inflammatory changes invisible to endoscopy. As our understanding of remission becomes increasingly multidimensional—encompassing mucosal, histological, and deeper wall healing—there is a growing need to determine how IUS can support or even refine these evolving targets. Future studies should therefore examine how IUS predicts deeper layers of healing, how its parameters correspond with histological outcomes, and whether IUS-defined transmural remission can serve as a meaningful endpoint within treat-to-target strategies for UC.

### 4.5. Segmental Disease Behavior

A broader, more nuanced understanding of UC pathology also reinforces the importance of carefully evaluating which bowel segments are assessed. Several included studies did not evaluate all colonic segments or did not analyze segment-level variation—an important limitation given evidence that UC healing can be patchy, with differing rates of mucosal recovery across locations [42,43]. De Voogd et al. further observed that IUS-assessed healing often progresses from proximal to distal segments, suggesting that averaging BWT across segments may obscure clinically meaningful variation [29,30,40]. Thus, segment-specific assessment likely improves both diagnostic and prognostic accuracy.

### 4.6. Establishing Meaningful Cut-Off Values

Given these biological and anatomical complexities, it is unsurprising that establishing universal and clinically meaningful BWT cut-offs remains challenging. The included studies varied widely in their methodological approaches: some advocated absolute thresholds (e.g., BWT ≤ 3 mm), while others favored relative changes (≥20% reduction). Ilvemark et al. proposed that percentage reduction offers a more generalizable and physiologically meaningful metric, as it accounts for baseline variability and reduces misclassification due to minor fluctuations in patients with high starting BWT [25]. Supporting this, both Ilvemark et al. and An et al. reported higher sensitivity for relative BWT reductions than absolute changes when predicting the need for rescue infliximab [25,26]. Conversely, De Voogd et al. found that absolute BWT thresholds outperformed relative reductions in predicting endoscopic outcomes, reflected in higher AUROC (area under the receiver operating characteristic curve) values and more favorable sensitivity and specificity [30]. These conflicting findings highlight an urgent need for methodological standardization in future IUS studies to enable consistent prognostic application.

### 4.7. Blinding of IUS Examiner

**Figure 2:**
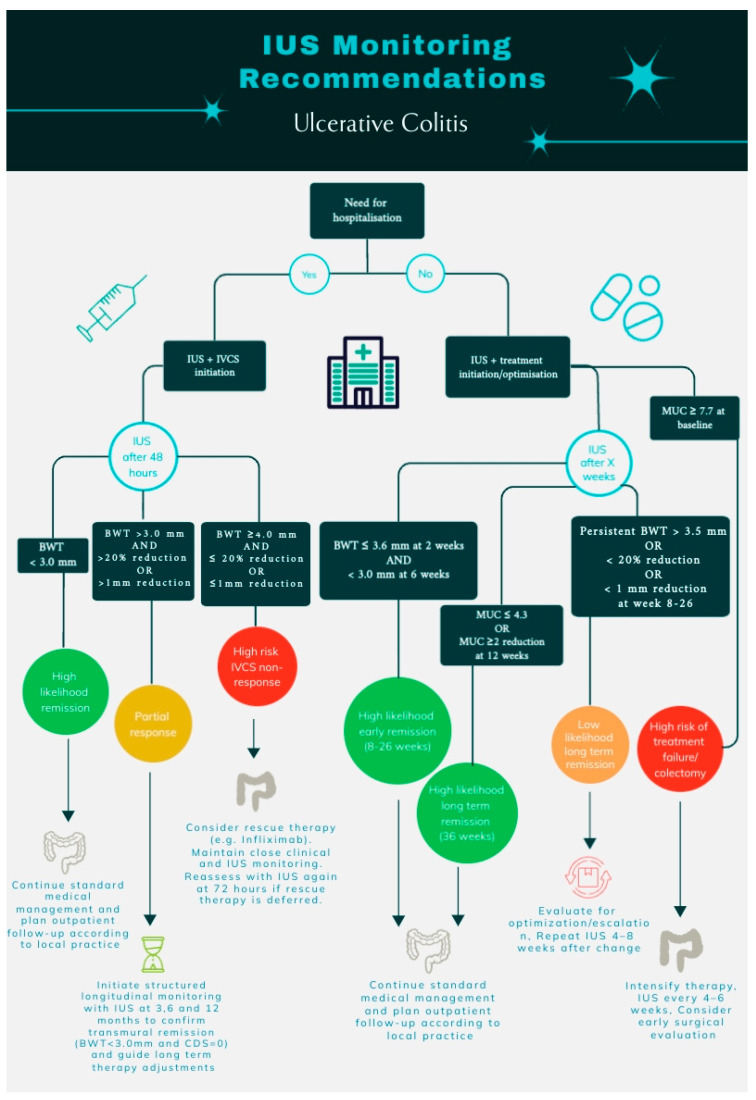
Recommendations for IUS monitoring of patients with ulcerative colitis. IUS, intestinal ultrasonography; BWT, bowel wall thickness; MUC, Milan Ultrasound Criteria; IVCS, intravenous corticosteroids; and CDS, color Doppler signal.

A noteworthy source of methodological heterogeneity across included studies related to the extent to which IUS examinations were blinded to patients’ clinical, biochemical, and endoscopic disease status. Among studies where such information was provided, IUS examiners were blinded in most studies; however, the degree of blinding varied (Figure 2). This variation has meaningful implications for interpreting the predictive accuracy of IUS parameters. Lack of blinding introduces the potential of expectation bias—an issue particularly relevant for semi-quantitative features such as CDS, BWS, haustration, and I-fat, where subjective interpretation may be influenced by prior clinical knowledge. While BWT is a more objective measurement and thus is less susceptible to bias, knowledge of clinical severity may influence which bowel segment is measured, probe pressure, or how measurements are repeated. Therefore, heterogenicity in blinding may partly explain variations in the reported findings among studies in this review.

### 4.8. Strengths of This Review

This review provides a comprehensive synthesis of IUS prognostication in UC, uniquely spanning both acute severe disease in hospitalized patients and longitudinal monitoring in outpatients. Its strengths include a prospectively registered protocol, a comprehensive dual-database search, and strict inclusion of prospective studies, ensuring a higher level of evidence than prior mixed-design reviews. Methodological rigor was reinforced through duplicate screening, independent data extraction, and a QUADAS-2 assessment specifically adapted to prognostic IUS research. By evaluating both individual IUS parameters and composite indices, the review offers a detailed comparison of their predictive performance and distills clinically meaningful thresholds across multiple outcomes. Importantly, these findings are translated into practical, scenario-specific recommendations that support real-world integration of IUS within treat-to-target care pathways.

### 4.9. Limitations of This Review

Despite its breadth, this review has several limitations. Methodological heterogeneity—including variation in IUS timing, segmental assessment, outcome definitions, and cut-off criteria—precluded meta-analysis and limits the generalizability of pooled conclusions. Importantly, these inconsistencies likely influence the strength and interpretation of the findings, as differences in ultrasound technique and anatomical segments analyzed reduce direct comparability across studies and hinder the identification of universally applicable thresholds. Variation in the timing of IUS assessments relative to treatment initiation is particularly relevant, as early treatment-induced changes—especially in bowel wall thickness—appear to carry distinct prognostic significance compared with baseline measurements. Likewise, heterogeneity in outcome definitions may partly explain why certain IUS parameters demonstrate strong predictive performance in some studies but not in others.

Several included studies were available only as conference abstracts, restricting access to detailed methodology and potentially relevant data, thus limiting the robustness of risk-of-bias assessment. The absence of full methodological details—in particular, incomplete reporting on patient selection procedures, blinding of IUS assessors, outcome adjudication, and handling of missing data resulted in multiple QUADAS-2 domains being rated as unclear. Such limitations may inflate apparent predictive performance—especially for operator-dependent parameters such as CDS and composite scores—or, conversely, obscure true associations. Although abstract-only studies were included to minimize selective reporting bias and capture emerging data in this rapidly evolving field, their inclusion increases uncertainty around some estimates and necessitates cautious interpretation of the overall conclusions.

Small sample sizes across multiple studies further reduced statistical power, likely contributing to non-significant findings despite clear numerical trends. Ilvemark et al., for example, observed that lower BWT cut-offs and early dynamic reductions remained statistically significant only at the cohort level, implying underpowered subgroup analyses [24].

Variation in examiner blinding represents another key limitation. Some studies blinded IUS assessors to clinical and endoscopic information, whereas others did not. Because sonographic interpretation can be influenced by prior clinical knowledge, unblinded assessments may overestimate the predictive accuracy of parameters such as CDS and composite scores. This inconsistency likely contributes to differences in reported performance across studies. Taken together, while heterogeneity limits precision and generalisability, the repeated identification of early BWT changes as a prognostic marker across diverse study designs supports the robustness of this specific finding, whereas conclusions regarding more complex or operator-dependent parameters should be interpreted with greater caution. These limitations underscore the need for larger, multicenter, methodologically harmonized studies to validate optimal IUS thresholds and improve reliability.

Finally, this review did not evaluate the comparative predictive value of IUS against established clinical, biochemical, or endoscopic markers. Understanding how IUS integrates within a multimodal monitoring framework is essential for its translation into routine clinical practice, and future research should prioritize such comparative analyses.

## 5. Conclusions

This systematic review indicates that IUS holds considerable predictive promise, with BWT emerging as the most robust and reproducible parameter across settings. Nevertheless, the evidence base is heterogeneous, and key questions remain regarding optimal BWT cut-offs, timing of assessments, and the incremental value of composite scores versus single parameters. Many reported thresholds have not yet been independently validated and may be influenced by operator experience, image acquisition, and study-specific definitions. Accordingly, the IUS-based thresholds and monitoring strategies outlined in this review should be viewed as hypothesis-generating rather than definitive clinical standards. Larger, prospective, multicenter studies with standardized protocols and independent validation are warranted to define clinically actionable thresholds and to quantify the added value of IUS over established clinical, biochemical, and endoscopic indices. Within these limitations, we propose pragmatic, evidence-informed frameworks for IUS-guided monitoring in both hospitalized and outpatient settings (Figure 2), intended to support future validation and refinement rather than immediate universal implementation. 

In the context of treat-to-target care and the STRIDE II framework [4]—which prioritizes clinical, biochemical, and endoscopic targets across immediate to long-term horizons—early IUS changes, particularly reductions in BWT, demonstrate strong potential as an adjunct target to guide therapy.

Overall, with standardized implementation and validation, IUS has the potential to improve risk stratification, enable earlier treatment optimization, and meaningfully advance personalized care in UC.

## Data Availability

Data are presented in the current manuscript and its Supplementary Materials.

## Supplementary Materials

The following supporting information can be downloaded at: https://www.mdpi.com/article/10.3390/jcm15010035/s1, Table S1: Literature search; Table S2: QUADAS-2 Guiding questions; Table S3: QUADAS-2 risk of bias assessment; Table S4: Study Characteristics; Table S5: Evidence Table. Hospitalized patients; Table S6: Evidence Table. Outpatients; Table S7: PRISMA Checklist.
