# Supplementary material for: Transabdominal Intestinal Ultrasonography in Monitoring and Predicting Outcomes in Ulcerative Colitis—A Systematic Review
Source: J Clin Med. 2025 Dec 20;15(1):35. doi: 10.3390/jcm15010035 (PMC12787012; doi:10.3390/jcm15010035)
Supplement: Supplementary file 1 [file jcm-15-00035-s001.zip › jcm-4048839-supplementary.pdf]

Supplementary Table S1: Literature search

**PubMed**	(colitis[tiab] OR "ulcerative colitis"[tiab]) AND ("intestinal ultrasound"[tiab] OR "bowel ultrasound"[tiab] OR "transabdominal ultrasound"[tiab] OR ultrasound[tiab] OR ultrasonic[tiab] OR sonographic[tiab] OR sonography[tiab] OR SICUS [tiab]) AND (prognosis[tiab] OR predictive[tiab] OR prediction[tiab] OR predict[tiab] OR response[tiab] OR respond[tiab] OR remission[tiab] OR relapse[tiab] OR prognostic[tiab] OR loss of response[tiab] OR discontinuation[tiab] OR persistence[tiab])
**Embase**	(colitis:ti,ab OR "ulcerative colitis":ti,ab) AND ("intestinal ultrasound":ti,ab OR "bowel ultrasound":ti,ab OR "transabdominal ultrasound":ti,ab OR ultrasound:ti,ab OR ultrasonic:ti,ab OR sonographic:ti,ab OR sonography:ti,ab OR SICUS:ti,ab) AND (prognosis:ti,ab OR predictive:ti,ab OR prediction:ti,ab OR predict:ti,ab OR response:ti,ab OR respond:ti,ab OR remission:ti,ab OR relapse:ti,ab OR prognostic:ti,ab OR "loss of response":ti,ab OR discontinuation:ti,ab OR persistence:ti,ab)

Literature search: MeSH and free text terms used.

Supplementary Table S2: QUADAS-2 Guiding questions

Domain	Example guiding question	Judgement
**1. Patient selection**	Were inclusion/exclusion criteria clearly defined? Was recruitment consecutive/prospective? Were UC diagnosis and baseline IUS performed before treatment initiation?	Low / High / Unclear
**2. Index test (IUS)**	Was IUS assessed and interpreted blinded to clinical or endoscopic outcomes? Were thresholds (e.g., BWT cut-off) pre-specified?	Low / High / Unclear
**3. Reference standard**	Was treatment response/remission defined using accepted criteria (clinical/endoscopic/biochemical)? Was the definition applied consistently?	Low / High / Unclear
**4. Flow and timing**	Was the interval between baseline IUS and outcome assessment appropriate? Were all included patients analyzed? Any loss to follow-up or missing data reported?	Low / High / Unclear
**Applicability concerns**	Are patients, IUS technique, and outcomes relevant to your review question (i.e., adults with UC, outpatient setting, standard medical therapy)?	Low / High / Unclear

QUASDAS-2 (Quality Assessment of Diagnostic Accuracy Studies) guiding questions

Supplementary Table S3: QUADAS-2 risk of bias assessment

Study ID / Citation	Patient Selection - R1	Patient Selection - R2	Patient Selection - Consensus	Index Test (IUS) - R1	Index Test (IUS) - R2	Index Test (IUS) - Consensus	Reference Standard - R1	Reference Standard - R2	Reference Standard - Consensus	Flow & Timing - R1	Flow & Timing - R2	Flow & Timing - Consensus	Applicability - R1	Applicability - R2	Applicability - Consensus
«Sonorescue»-treatment response in acute severe Ulcerative Colitis measured with ultrasound (Nylund, 2025)	Low	Low	Low	Unclear	Unclear	Unclear	Low	Low	Low	Low	Low	Low	Low	Low	Low
Baseline Hypertrophy of the Submucosa at intestinal ultrasound predicts Failure of Treatment in patients with ulcerative colitis (de Voogd, 2024)	Low	Low	Low	Unclear	Unclear	Unclear	Low	Low	Low	Low	Low	Low	Low	Low	Low
EARLY ASSESSMENT WITH GASTROINTESTINAL ULTRASOUND IN PATIENTS HOSPITALISED FOR A FLARE OF ULCERATIVE COLITIS AND PREDICTING THE NEED FOR	Low	Low	Low	Low	Low	Low	Low	Low	Low	Low	Low	Low	Low	Low	Low

SALVAGE THERAPY: A PILOT STUDY (Smith, 2021)															
Early Intestinal Ultrasound in Severe Ulcerative Colitis Identifies Patients at Increased Risk of 1-Year Treatment Failure and Colectomy (Ilvemark, 2024)	Low	Low	Low	Low	Low	Low	Low	Low	Low	Low	Low	Low	Low	Low	Low
Early Intestinal Ultrasound Predicts Clinical and EndoscopicTreatment Response and Demonstrates Drug- Specific Kinetics in Moderate-to-Severe Ulcerative Colitis (de Voogd, 2023)	Low	Low	Low	Low	Low	Low	Low	Low	Low	High	Low	Low	Low	Low	Low
Early Intestinal Ultrasound Predicts Long-Term Endoscopic Response to Biologics in Ulcerative Colitis (Allocca, 2023)	Low	Low	Low	Low	Low	Low	Low	Low	Low	Low	Low	Low	Low	Low	Low

Gastrointestinal ultrasound predicts need for rescue therapy in a multicenter prospective cohort of patients with acute severe ulcerative colitis: USUC study, including the GENIUS cohort (Y-K AN, 2024)	Low	Low	Low	Low	Low	Low	Low	Low	Low	Low	Low	Low	Low	Low	Low
Intestinal ultrasound as a prognostic tool in new-onset ulcerative colitis—a Copenhagen IBD Cohort Study (Madsen, 2025)	Low	Low	Low	Low	Low	Low	Low	Low	Low	Low	Low	Low	Low	Low	Low
Intestinal Ultrasonography predicts short term and clnical remission in patients with ulcerative colitis (Zorzi, 2021)	Low	Low	Low	Unclear	Unclear	Unclear	Low	Low	Low	Low	Low	Low	Low	Low	Low
Monitoring of vedolizumab therapy using fecal calprotectin, abdominal ultrasound, and drug levels in ulcerative colitis: a predictive	Unclear	Unclear	Unclear	Low	Low	Low	Low	Low	Low	Low	Low	Low	Low	Low	Low

algorithm (Helwig, 2025)															
Predictive value of Milan ultrasound criteria in ulcerative colitis: A prospective observational cohort study (Allocca, 2022)	Low	Low	Low	Low	Low	Low	Low	Low	Low	Low	Low	Low	Low	Low	Low
Prognostic value of intestinal ultrasound (IUS) in patients with Acute Severe Ulcerative colitis (ASUC) – A single center experience (Zacharopoulou, 2025)	Low	Low	Low	Unclear	Unclear	Unclear	Low	Low	Low	Low	Low	Low	Low	Low	Low
Quantitative contrast-enhanced ultrasound for monitoring vedolizumab therapy in inflammatory bowel disease patients: a pilot study (Goertz, 2018)	Low	Low	Low	Low	Low	Low	Low	Low	Low	Low	Low	Low	Low	Low	Low

Submucosal hyperechogenicity on intestinal ultrasound is associated with fat deposition and is a predictor of endoscopic non-response to anti-inflammatory treatment in Ulcerative Colitis (Pruijt, 2025)	Unclear	Unclear	Unclear	Unclear	Unclear	Unclear	Low	Low	Low	Low	Low	Low	Low	Low	Low
Superior predictive value of transmural over endoscopic severity for colectomy risk in ulcerative colitis: a multicentre prospective cohort study (Seda, 2024)	Low	Low	Low	Low	Low	Low	Low	Low	Low	Low	Low	Low	Low	Low	Low
The role of transabdominal ultrasound in evaluating Ulcerative Colitis disease activity and predicting treatment response (Peng, 2024)	Low	Low	Low	Low	Low	Low	Low	Low	Low	Low	Low	Low	Low	Low	Low

Transabdominal ultrasonography to assess intestinal wall thickness and vascularity appears to predict therapeutic effects of steroid treatment in moderate to severe ulcerative colitis patients (Ogashiwa, 2015)	Low	Low	Low	Unclear	Unclear	Unclear	Low	Low	Low	Unclear	Unclear	Unclear	Low	Low	Low
Very early IUS predicts IVCS failure in severe UC (Ilvemark, 2022)	Low	Low	Low	Low	Low	Low	Low	Low	Low	High	Low	Low	Low	Low	Low

QUADAS-2 (Quality Assessment of Diagnostic Accuracy Studies), risk of bias assessment of included studies

Supplementary Table S4: Study Characteristics

Reference (name + year + first author)	Study design	Number of participants (UC)	Disease extent/severity at baseline	In hospital / outpatient	Female (%)	Age at inclusion (mean ±SD / median [IQR])	Disease duration years (mean ±SD / median [IQR])	IUS timing and parameters (when + which)	IUS examiners and blinding	Previous treatments	Current treatments	Treatment investigated in relation to IUS	Follow-up (time)	Outcome definition (incl. cut-offs)
**Baseline Hypertrophy of the Submucosa at intestinal ultrasound predicts Failure of Treatment in patients with ulcerative colitis (de Voogd, 2021)**	Prospective cohort (poster)	49	EMS ≥2 Extending beyond rectum	Outpatient	N/A	N/A	N/A	Baseline. Follow up between week 8-26<br><br>Sigmoid colon was assessed for: BWT, individual wall thickness (mucosa, submucosa, muscular propria), ratio between layers, CDS, loss of haustrations, loss of stratification, HoS	N/A	61% failed ≥1 biological	N/A	59% started tofacitinib<br>41% started a biological	8–26 weeks	Endoscopic remission (ER). Defined as EMS=0 in sigmoid colon Endoscopic improvement (EI). Defined as EMS≤1 in sigmoid colon
**Early assessment with gastrointestinal ultrasound in patients hospitalized for a flare of ulcerative colitis and predicting the need for salvage therapy: a pilot study (Smith, 2021)**	Prospective pilot (full text)<br>Single center	10	Severe UC (> 6 bowel actions per day)<br>E2: 4<br>E3: 2	In hospital	10,0%	30 (21-39) years	10 (1-19) years	Baseline (within 24 hours of hospital admission), day 3 and day 7<br><br>BWT in rectum, sigmoid, descending, transverse, ascending colon., CDS, Limberg score, wall stratification assessment, loss of haustration and presence or absence of	Sonographic assessment was performed by two independent gastroenterologists, blinded to each other's findings. Also, these assessments were performed blinded to the clinical progress and decisions regarding patients	N/A	No medical therapy: 6<br>5 ASA: 4<br>Thiopurine: 1<br>Oral corticosteroids: 2<br>Infliximab 1	IV corticosteroids (100 mg x 4 daily)	Day 3 and 7	Need for rescue infliximab on day 3 if: ≥ 8 stools/day, or ≥ 3 stools/day and CRP > 45 mg/L<br>Need for rescue infliximab on day 7 if: ≥ 3 stools/day with visible blood

								lymphadenopathy or mesenteric hyperechogenicity.						
**Early Intestinal Ultrasound in Severe Ulcerative Colitis Identifies Patients at Increased Risk of 1-Year Treatment Failure and Colectomy (Ilvemark, 2024)**	Prospective cohort<br>3 centers (full text)	56	Severe UC: Mayo score ≥ 8 AND BWT ≥ 3 mm in the sigmoid colon<br><br>E1: 0 [0]<br>E2: 9 [16]<br>E3: 47 [84]<br><br>EMS 1: 3 [5]<br>EMS 2: 22 [39]<br>EMS 3: 31 [56]<br>pMayo: 10 (8-12)	In hospital	48%	35.5 (18-70) years	1.0 (0-33)	Baseline; 48±24 hours; 6±1 days; 3 months<br><br>BWT, CDS (assessed by Limberg score), BWS, inflammatory mesenteric fat [i-fat], and haustration	Sonographic assessment was performed by 7 IUS examiners - all blinded to non-IUS parameters of disease throughout the study period, baseline being the only assessment. The treating physician was blinded to all IUS results	No previous medication: 22 (39%)<br>IV corticosteroids 11 (20%)<br>Oral corticosteroids 18 (32%)<br>Local corticosteroids 3 (5%)<br>Oral 5-ASA 27 (48%)<br>Local 5-ASA 25 (44%)<br>AZA/MP6 3 (5%)<br>Infliximab 3 (5%)<br>Vedolizumab 2 (3%)<br>Ustekinumab 0 (0%)<br>Adalimumab 0 (0%)<br>Golimumab 1 (2%)	None 23 (41%)<br>Oral steroids 7 (12%)<br>Local steroids 2 (3%)<br>Oral 5-ASA 29 (52%)<br>Local 5-ASA 8 (14%)<br>AZA/MP6 2 (3%)	IV corticosteroids	3 and 12 months	At 3 and 12 months after IV corticosteroids<br>Any intervention, defined as increase in corticosteroid dosing during tapering, re-initiation of corticosteroids, start or shift in biologics, and/or hospitalization due to flare in UC and/or colectomy. During 12-month follow-up<br><br>Colectomy<br><br>Endoscopic

															Remission: MES = 0-1 at 3 months
**Early Intestinal Ultrasound Predicts Clinical and Endoscopic Treatment Response and Demonstrates Drug- Specific Kinetics in Moderate-to-Severe Ulcerative Colitis (de Voogd, 2023)**	Prospective longitudinal (full text) Single center	51	Moderate to severe UC defined as: EMS ≥2 AND IUS with disease activity in at least 1 segment (BWT >3.0mm and 1 additional pathological parameter (color Doppler signal [CDS], loss of haustrations, loss of wall layer stratification,	Outpatient	55%	38 (18-72)	N/A	Baseline, W2, W6, W8–26<br><br>All colonic segments: BWT, CDS, Haustrations, BWS, fatty wrapping, mesenteric lymph nodes	The IUS examiner was blinded to biochemical and endoscopic disease activity.<br>A second reader (also blinded for clinical, biochemical and endoscopic data), read 30 randomly selected IUS examinations and scored all IUS parameters.	Corticosteroids 47 (92%)<br>Aminosalicylates 51 (100%)<br>Thiopurines 32 (63%)<br>Methotrexate 5 (10%)<br>Previous biological and/or JAK inhibitor exposure<br>0 : 24 (47%)<br>1 : 12 (24%)<br>2 : 15 (29%)	N/A	Systemic corticosteroids 40 (78)<br><br>Aminosalicylates:<br>Monotherapy 2 (4)<br>Combination therapy 29 (57)<br><br>Thiopurines:<br>Monotherapy 3 (6)<br>Anti-TNF combination therapy 15 (29)<br><br>Biologics:<br>Infliximab 18 (35)<br>Adalimumab 1 (2)<br>Vedolizumab 14 (27)<br>Ustekinumab 2 (4)	IUS examinations: baseline, W2, W6, and W8 to W26<br><br>SCAAI score: baseline, W2, W6, and between W8 and W26<br><br>Endoscopy: baseline, between W8-W26	Endoscopic remission: EMS = 0, Endoscopic improvement: EMS ≤1, Endoscopic response: EMS decrease ≥1. Complete endoscopic remission and improvement: EMS = 0 or ≤1<br><br>Primary outcome: If BWT as measured by IUS is a surrogate

			fatty wrapping, or mesenteric lymph nodes) or a BWT ≥5.0 mm in the rectum<br><br>E2: 25 (49)<br>E3: 26 (51)									Tofacitinib 14 (27)<br>Ciclosporin 1 (2)		marker of endoscopic improvement after 6 weeks of treatment.<br>(Ie Week 6 BWT ≤3.0 mm → predicts remission)
**Early Intestinal Ultrasound Predicts Long-Term Endoscopic Response to Biologics in Ulcerative Colitis (Allocca, 2023)**	Prospective observational cohort (full text)<br>Single center	49	EMS 2: 10 [20]<br>EMS 3: 39 [80]<br>E2: 33 [67]<br>E3: 16 [33]	Outpatient	31%	N/A	5.8 (2.8-10.8)	Baseline, week 12, reassessment (mean 9.4 ± 3.6 months)<br><br>MUC	1 IUS examiner performed all IUS exams, blinded to patient's symptoms and endoscopic findings.	Steroid 26<br>Azathioprine 21<br>Biologics 24	N/A	Infliximab: 29 (59%)<br>Adalimumab: 4 (8%)<br>Vedolizumab: 14 (29%)<br>Ustekinumab: 2 (4%)<br>Steroids (concomitant): 23<br>Azathiopurine (concomitant): 15	≤1 year	Whether ultrasound improvement [MUC ≤ 6.2] at week 12, during biologic therapy, could predict endoscopic improvement [MES ≤ 1] at reassessment<br><br>to evaluate whether ultrasound improvement at

														week 12, during biologic therapy, could predict endoscopic remission at reassessment [MES = 0];<br><br>to investigate the minimum improvement in MUC able to predict endoscopic improvement and remission at reassessment;
**Early Intestinal Ultrasound Predicts Intravenous Corticosteroid Response in Hospitalized Patients With Severe Ulcerative Colitis (Ilvemark, 2022)**	Prospective. Multicenter (3 centers) (full text)	56	Severe UC: Mayo score ≥ 8 AND BWT ≥ 3 mm in the sigmoid colon<br><br>E1: 0 [0]<br>E2: 9 [16]<br>E3: 47 [84]<br><br>EMS 1: 3 [5]<br>EMS 2: 22 [39]<br>EMS 3: 31 [56]<br>pMayo: 10 (8-12)	In hospital	48%	35.5 (18-70) years	1.0 (0-33)	Baseline; 48±24 hours; 6±1 days; 3 months<br><br>BWT, CDS (assessed by Limberg score), BWS, inflammatory mesenteric fat [i-fat], and haustration	At baseline all 7 IUS examiners were aware that the patient was admitted to hospital due to severe UC, but they were blinded during the study period. Treating and recruiting physicians were blinded to all IUS results.	No previous medication: 22 (39%)<br>IV corticosteroids 11 (20%)<br>Oral corticosteroids 18 (32%)<br>Local corticosteroids 3 (5%)<br>Oral 5-ASA 27 (48%) Local 5-ASA 25 (44%)<br>AZA/MP6 3 (5%)<br>Infliximab 3 (5%)<br>Vedolizumab 2 (3%)<br>Ustekinumab 0 (0%)<br>Adalimumab 0 (0%)<br>Golimumab 1 (2%)	None 23 (41%) Oral steroids 7 (12%) Local steroids 2 (3%)<br>Oral 5-ASA 29 (52%)<br>Local 5-ASA 8 (14%)<br>AZA/MP6 2 (3%)	IV corticosteroids	7 days	Treatment response: pMayo at day 6±1: ≥30% and ≥3 point reduction with either a rectal bleeding subscore of 0 or 1 OR a decrease in rectal subscore ≥1 point.<br><br>Treatment failure: need for rescue therapy

**Reduction in bowel wall thickness and Doppler signals as early as 36 hours predicts corticosteroid response in a multi-center prospective cohort of Acute Severe Ulcerative Colitis: USUC Study including the GENIUS cohort (An, 2023)**	Prospective observational cohort study 5 centers (abstract)	25	ASUC	In hospital	60%	Mean 34.1 years	Mean 4.8 years	Baseline (SV1, admission day), day 3 (SV2), and at hospital discharge (SV3), and thereafter as an outpatient at 1 month (SV4), 4 months (SV5), and 10 months (SV6) after discharge<br><br>BWT, CDS for each bowel segment	N/A	Biological exposure 20%	N/A	IV corticosteroids	N/A	IV steroid therapy failure on day 3 = need for rescue therapy (IFX)
**Intestinal ultrasound as a prognostic tool in new-onset ulcerative colitis—a Copenhagen IBD Cohort Study (Madsen, 2025)**	Prospective cohort 2 centers (full text)	193	E1: 0<br>E2: 85 (44)<br>E3: 108 (56)<br><br>EMS 1: 41 (21.2)<br>EMS 2: 99 (51.3)<br>EMS 3: 36 (18.7)	Outpatient	46%	43 (28-57) years	0 years	Baseline (within median time of 15 days from diagnostic endoscopy), 3 months and 12 moths<br>BWT, CDS, BWS, i-fat for all colon segments. IBUS-SAS score calculated for the worst-affected segment.	9 IUS examiners were not blinded to the patient journal or other examinations	None	Steroid (median duration 5 days): 44 (30%)	Corticosteroids, Biologics	12 months	Colectomy or need for systemic steroids during follow-up period (1 year after diagnosis)<br><br>Steroid-free clinical remission at 3, 6, 9, and 12 months after diagnosis

**Intestinal Ultrasonography predicts short term clinical remission in patients with moderate to severe ulcerative colitis (Zorzi, 2021)**	Prospective cohort (abstract)	31	Mod-severe Total mayo 6-10: 84% Total mayo >10: 16% E2: 16%, E3: 84%	Outpatient	61%	45 (18-7) years	9 (0.25–26) years	Baseline.BWT, echopattern, blood flow (adapted Limberg' score), MUC, shear wave elastography values	N/A	N/A	N/A	Anti-TNFs 48%Vedolizumab 39% Ustekinumab 10% Tofacitinib 3%	3 months	Clinical remission at 3 months was defined as partial Mayo Score=0-1
**Monitoring of vedolizumab therapy using fecal calprotectin, abdominal ultrasound, and drug levels in ulcerative colitis: a predictive algorithm (Helwig, 2025)**	Prospective (abstract)	17	UC not more precisely specified	Outpatient	N/A	N/A	N/A	Baseline, week 6 BWT	N/A	N/A	N/A	Vedolizumab	98	Clinical response or remission at week 14 or 22 defined as: vedolizumab ≥ 24 µg/ml, ≥ 25% reduction in colon wall thickness, or ≥ 50% reduction in feacal calprotectin

**Predictive value of Milan ultrasound criteria in ulcerative colitis: A prospective observational cohort study (Allocca, 2022)**	Prospective observational cohort. Single center (full text)	98	E1: 8 (8)<br>E2: 44 (45)<br>E3: 46 (47)<br><br>EMS 0–1: 31 (32)<br>EMS 2-3: 67 (68)	Outpatient	38%	N/A	9.4 (22.1-44.5) years	Baseline BWT. For each affected intestinal segment, a mean of two measurements was calculated. BWS (graded 0–3: normal to lost) CDS (0 = absent, 1 = present on Doppler), Stricture (wall thickening with luminal narrowing ± proximal dilatation) Fistula (hypoechoic tract ± hyperechoic content), Abscess (round anechoic lesion with irregular wall, no blood flow) Mesenteric lymph nodes (presence/absence) Mesenteric hypertrophy (presence/absence). The worst-affected segment was used for analysis.	2 gastroenterologists performed IUS and measured MUC, blinded to patients' symptoms as well as clinical and endoscopic scores.	Steroids 32<br>Immunosuppressants 32<br>Biologics 19	Steroids 22<br>Immunosuppressants 4<br>Biologics 24	Steroids 22<br>Immunosuppressants 4<br>Biologics 24	1.6 (0.9-2.7) years	UC-related negative course: defined as the need for corticosteroids, treatment escalation, hospitalization, or need for colectomy

**Prognostic value of intestinal ultrasound (IUS) in patients with Acute Severe Ulcerative colitis (ASUC) – A single center experience (Zacharopoulou, 2025)**	Prospective. Single center (abstract)	6	ASUC	In hospital	17%	73 ± 11 years	6.8 ± 8.6 years	Baseline (≤48 h post-IVCS)<br><br>BWT; modified Limberg score, loss of bowel stratification in LC, TC, RC; free fluid in peritoneal cavity, pneumatosis intestinal, presence of mesenteric hypertrophy, enlarged lymph nodes	N/A	N/A	N/A	IV corticosteroids	N/A	Treatment failure: need for rescue therapy or need for urgent colectomy
**Quantitative contrast-enhanced ultrasound for monitoring vedolizumab therapy in inflammatory bowel disease patients: a pilot study (Goertz, 2018)**	Prospective observational pilot (full text)	7	IBD (UC 7, CD 11)<br>Mayo clinical subscore 5.1 (+/- 1.7)	Outpatient	17%	51 +/-9 years	7.3 +/-6 years	Baseline and week 14 (CEUS)<br>A range of CEUS perfusion parameters (amplitude- and time-derived) to monitor microvascularization changes in the bowel wall during vedolizumab therapy.<br>Only the most affected bowel wall segment was assessed.	3 unblinded IUS examiners	Biologics 71.4%	N/A	Vedolizumab induction	14 weeks	Clinical response vs. non-response at week 14. Defined as clinical mayo subscore Δ ≥ -1

**«Sonorescue»-treatment response in acute severe Ulcerative Colitis measured with ultrasound (Nylund, 2025)**	Prospective, two-center (abstract)	64	ASUC<br>E2-E3<br>EMS ≥ 2	In hospital	N/A	N/A	N/A	Baseline. Follow up day 2–3 BWT, CDS, loss of haustration and fatty tissue reaction, UC-ultrasound index score	N/A	N/A	N/A	IV corticosteroids	2–3 days in-hospital	Need for rescue Infliximab treatment (clinical decision) Lindgren's criteria for colectomy
**Submucosal hyperechogenicity on intestinal ultrasound is associated with fat deposition and is a predictor of endoscopic non-response to anti-inflammatory treatment in Ulcerative Colitis (Pruijt, 2025)**	Prospective longitudinal validation cohort (full text)	47	UC<br>E1: 2 (4%)<br>E2: 23 (49%)<br>E3: 22 (47%)<br><br>EMS 0: 2 (4%)<br>EMS 1: 1 (2%)<br>EMS 2: 20 (44%)<br>EMS 3: 22 (49%)	Outpatient	25 (53%)	37.0 (27-51) years	7.0 (3-11) years	Baseline Relative Submucosal Echogenicity (RSE), BWT, CDS, loss of stratification, loss of haustration, presence of lymph nodes	1 ultrasonographer reviewed all IUS images - blinded from clinical, biochemical and pathology data. No information was provided about IUS examiners.	Failed >/= 2 biologics: 19 (41%)	N/A	Infliximab: 9 (19%)<br>Vedolizumab: 6 (13%)<br>Ustekinumab 1 (2%)<br>Tofacitinib: 29 (62%)<br>Systemic corticosteroids: 2 (4%)	9.6 (8.7-20.4) weeks	Endoscopic response after 8-26 weeks: defined as ≥1 point decrease in EMS
**Superior predictive value of transmural over endoscopic severity for colectomy risk in ulcerative colitis: a multicenter prospective cohort study (Sed, 2024)**	Prospective time-to-event<br>Multicenter (2 centers)<br>Full text	141	EMS 0: 24 [17%]<br>EMS 1: 22 [15.6%]<br>EMS 2: 29 [20.6]<br>EMS 3: 66 [46.8%]<br><br>PMS ≥ 2 :77 [55%]<br><br>E1: 8 [5.7%]<br>E2: 72 [51.1%]<br>E3: 61 [43.3%]	Outpatient	39.7%	46.5 (33.1–60.4) years	9 (4–16.8) years	Baseline MUC	IUS examiners were blinded to clinical and endoscopic scores	N/A	Steroids 38 [26.9%]<br>Immunosuppressant 8 [5.7%]<br>Biological therapy 35 [24.8%]	N/A	1.8 [1.0–2.7] years	Colectomy

**The role of transabdominal ultrasound in evaluating Ulcerative Colitis disease activity and predicting treatment response (Peng, 2024)**	Prospective cohort Single center (abstract)	80 (315 colon segments)	UC mod-severe	Outpatient	N/A	N/A	N/A	Baseline and post-induction (not specified when) In SC and DC: BWT, CDS, WLS, fatty wrapping, presence of lymph nodes	N/A	N/A	N/A	Treatment induction (not specified which)	After treatment induction (duration not specified)	Endoscopic remission: MES=0-1 or UCEIS=0-1 Endoscopic response: decrease in MES ≥1 or a reduction in UCEIS ≥2.
**Transabdominal ultrasonography to assess intestinal wall thickness and vascularity appears to predict therapeutic effects of steroid treatment in moderate to severe ulcerative colitis patients (Ogashiwa, 2015)**	Prospective (abstract)	100	Moderate to severe	In hospital	N/A	N/A	N/A	Baseline Main lesion: BWT, CDS	N/A	N/A	N/A	IV corticosteroids	60 days	Treatment failure (defined as need for Colectomy, or need for second line therapy: Calcineurin inhibitors and anti-tumor necrosis factor (TNF) therapy) within 60 days

Table of study characteristics **(Disease Extent** (Montreal Classification): **E1**: Proctitis**, E2**: Left-sided colitis**, E3**: Extensive colitis**. Colonic Segments: SC**: Sigmoid Colon**, DC**: Descending Colon**. Endoscopic Indices: MES**: Mayo Endoscopic Subscore**, EMS**: Endoscopic Mayo Score**, UCEIS**: Ulcerative Colitis Endoscopic Index of Severity. **Clinical Scores: PMS**: Partial Mayo Score**, fMS**: Full Mayo Score**. Ultrasound Parameters, BWS**: Bowel Wall Stratification**, WLS**: Wall Layer Stratification**, BWT** Bowel Wall Thickness, **CDS**: Color Doppler Signal**, HoS**: Hyperechogenicity of the Submucosa**, RSE**: Relative Submucosal Echogenicity**, WiR**: Wall Inflammation Ratio**. Composite Ultrasound Scores: MUC**: Milan Ultrasound Criteria (MUC = 1.4 × BWT + 2 × CDS)**, IBUS-SAS**: International Bowel Ultrasound Segmental Activity Score)

Supplementary Table S5: Evidence Table. Hospitalized patients.

IV corticosteroids

Study	IUS parameter	IUS timing after treatment	Prognostic value	Definition of outcome	Follow-up time/when outcome measured
**Need for rescue therapy**					
**Nylund 2025**	BWT (SC)	2-3 days after IVCS initiation	AUROC 0.87 (0.79-0.96)	Clinical decision for IFX	3
	BWT (sum all segments)		AUROC 0.84 (0.75-0.94)		
	UC-Ultrasound Index score		AUROC 0.84 (0.74-0.94)		
**Smith 2021**	BWT (mean)	Baseline within 24 hours after hospital admission	Responder: 4.6 mm<br>Non-responder: 6.2 mm<br>p=0.009	Need for rescue IFX on day 3 if:<br>≥ 8 stools/day, or<br>≥ 3 stools/day and CRP > 45 mg/L<br><br>Need for rescue IFX on day 7 if:<br>≥ 3 stools/day with visible blood	7 days
	BWT (SC)		Responder: 5.0 mm<br>Non-responder: 7.0 mm<br>p=0.033		
**An 2023**	BWT (mean)	Baseline (admission day)	Responder: 6.1 mm<br>Non-responder: 6.3 mm<br>(No significant difference)	Need for rescue IFX on day 3	3 days
	BWT reduction	Baseline (admission day) vs. day 3	Responder: BWT decreased by 2.1 mm (95% CI, 1.3–2.9; p=0.003)<br><br>Non-responder: BWT decreased by 0.7 mm (95% CI, 0.7–1.7 mm))<br><br>p=0.11		

	Absolute BWT reduction <1,40mm		AUC 0.76. Sensitivity: 63%, Specificity 75%,		
	Relative BWT reduction <20%		AUC 0.78<br>Sensitivity 81%, Specificity 75%		
**Ilvemark 2022**	BWT	Baseline (before IVCS)	Responders vs. non-responders: no significant difference<br>p=0.66	Need for rescue treatment on day 7	7 days
	CDS present		Responders vs. non-responders: no significant difference<br>p=0.43		
	Ifat present		Responders vs. non-responders: no significant difference<br>p=0.74		
	Destratification of BW		Responders vs. non-responders: no significant difference<br>p=0.31		
	Loss of haustrations		More frequent in non-responders<br>p=0.014		
	BWT ≥4.0 mm	48 ± 24 hours after IVCS initiation	AUC 0.77 (95 % CI 0.71, 0.74)		
	BWT absolute reduction ≤1.0mm		AUC 0.71 (95% CI 0.56 - 0.86)		
	BWT relative reduction ≤20%		AUC 0.74 (95% CI 0.60 - 0.88)		
	CDS present		More prevalent in non-response group.<br>p=0.04		

	Destratification of BW		Responder vs. non-responder: no significant difference p=0.31		
	Ifat present		Responder vs. non-responder: no significant difference p=0.43		
	Loss of haustrations		More prevalent in response group p=0.01		
	CDS present	6 ± 1 days	Responder vs. non-responder: no significant difference p=0.31		
	Destratification of BW		Responder vs. non-responder: no significant difference p=1.0		
	Ifat present		Responder vs. non-responder: no significant difference p=1.0		
	Loss of haustrations		Responder vs. non-responder: no significant difference p=0.42		
**Risk of colectomy**					
**Nylund 2025**	BWT (SC)	2-3 days after IVCS initiation	AUROC 0.86 (0.75-0.96)	Lindgren score = high risk colectomy	3 days
	BWT (sum all segments)		AUROC 0.88 (0.80-0.97)		
	UC-Ultrasound Index score		AUROC 0.85 (0.76-0.94)		
**Ilvemark 2024**	BWT (SC) <3.0mm	48 ± 24 hours after IVCS initiation	0% colectomy after 1 year	Colectomy within 1 year follow-up	1 year

	BWT (SC) ≥4.0 mm	48 ± 24 hours after IVCS initiation	OR 9.5 (95% CI: 1.4–64)		
	BWT (persistent ≥3.5mm)	6 ± 1 days	OR 8.3 (95% CI: 1.7–40)		
**Ogashiwa 2015**	BWT ≥ 7.0 mm	Baseline (before IV steroids)	Log-rank p=0.075	Colectomy within 60-day follow-up	60 days
**Treatment failure**					
**Zacharapoulou 2025**	BWT (right colon)	Baseline (≤48 h after IVCS initiation)	Responder: 2.4 ± 0.3 mm<br>Non-responder: 6.3± 0.3 mm<br>p=0.0001	Need for rescue IFX or urgent colectomy	Not specified
**Ilvemark 2024**	BWT (SC) ≥3.0 mm	48 ± 24 hours after IVCS initiation	OR 3.6 (95% CI: 1.1–12)	Any intervention: increase in corticosteroid dosing during tapering, re-initiation of corticosteroids, start or shift in biologics, and/or hospitalization due to flare in UC and/or colectomy.	12 months
	BWT (persistent) ≥3.5 mm	6 ± 1 days	OR 3.9 (95% CI: 1.2–13)		
	BWT (persistent) ≥3 mm	3 months	HR 3.5 (95% CI: 1.4–8.9)		
**Ogashiwa 2015**	BWT ≥7.0 mm	Baseline (before IV steroids)	log-rank p=0.021	Need for second line therapy: Calcineurin inhibitors or anti-tumor necrosis factor (TNF) therapy)	60 days
	BWT		Responder: 6.7 ± 2.3 mm<br>Non-responder: 8.0 ± 2.2 mm<br>p=0.005	Need for second line therapy: Calcineurin inhibitors or anti-tumor necrosis factor (TNF) therapy)<br>AND/OR<br>colectomy	
	BWF		Responder: 1.6 ± 1.1<br>Non-responder: 2.2 ± 0.9<br>p=0.016		

**Endoscopic remission**					
**Ilvemark 2024**	BWT normalization (<3mm)	3 months	OR 12.5 (95% CI: 2.3–67)	MES 0-1 at 3 months	3 months
**Clinical non-response**					
**Ilvemark 2022**	BWT	Baseline (before IVCS)	Responder vs. non-responder - no significant difference	Non-response: pMayo at day 6±1: < 30% and <3 point reduction with either a rectal bleeding subscore >1 OR a decrease in rectal subscore <1 point.	7 days
	CDS present		Responder vs. non-responder: no significant difference p=1.0		
	Destratification of BW		Responder vs. non-responder: no significant difference p=0.99		
	Ifat present		Responder vs. non-responder: no significant difference p=0.24		
	Loss of haustrations		Responder vs. non-responder: no significant difference p = 0.074		
	BWT ≥4.0 mm	48 ± 24 hours after IVCS initiation	AUC 0.85 (95% CI 0.76 - 0.95)		
	BWT absolute reduction ≤1.0mm		AUC 0.81 (95% CI 0.69-0.93)		
	BWT relative reduction ≤20%		AUC 0.85 (95% CI 0.74 - 0.95)		
	BWS normal		More prevalent in response group p=0.01		

	Ifat absent		More prevalent in response group<br>p=0.003		
	Haustrations present		More prevalent in response group<br>p<0.01		
	CDS		Responder vs. non-responder - no significant difference<br>0.26		
	Haustrations present	6 ± 1 days	More prevalent in response group<br>p<0.001		
	BWS normal		Responder vs. non-responder: no significant difference<br>p=0.49		
	Ifat absent		Responder vs. non-responder: no significant difference<br>0.16		
	CDS		Responder vs. non-responder: no significant difference<br>p=0.59		

Evidence table for data concerning Ulcerative Colitis hospitalized Patients. **Colonic Segments**: **SC**: Sigmoid Colon, **DC**: Descending Colon**. Endoscopic Indices: MES**: Mayo Endoscopic Subscore**, EMS**: Endoscopic Mayo Score**, UCEIS**: Ulcerative Colitis Endoscopic Index of Severity. **Clinical Scores: pMayo score**: Partial Mayo Score**. Ultrasound Parameters, BWS**: Bowel Wall Stratification**, WLS**: Wall Layer Stratification**, BWT** Bowel Wall Thickness, **BWF:** Bowel Wall Flow. **CDS**: Color Doppler Signal**, HoS**: Hyperechogenicity of the Submucosa**, RSE**: Relative Submucosal Echogenicity**, WiR**: Wall Inflammation Ratio**. Composite Ultrasound Scores: MUC**: Milan Ultrasound Criteria (MUC = 1.4 × BWT + 2 × CDS). **IBUS-SAS:** International Bowel Ultrasound Segmental Activity Score.

Supplementary Table S6: Evidence Table. Outpatients.

Study	IUS parameter /predictor	IUS timing after treatment	Prognostic value	Definition of outcome	Outcome timing / follow up period
Endoscopic remission					
De Voogd 2021	HoS	Baseline (prior to starting treatment)	Obs lack of ER:<br>OR 0.10 (95% CI: 0.01–0.87, p=0.014)	EMS=0 in SC	8-26 weeks
De Voogd 2023	BWT reduction	Baseline vs. week 8-26	2.32 ± 1.63 to 1.00 ± 1.98 mm (p=0.034)		
	BWT (SC) (threshold ≤3.0 mm)	Week 6	AUROC 0.82 (95% CI 0.63–1.00, p=0.007)<br>OR 25.13 (95% CI 2.01–314); p=0.012		
	BWT (DC) (threshold ≤3.20 mm)	Week 6	AUROC 0.89 (95% CI 0.74–1.00, p=0.003)		
	Submucosal thickness, pr mm increase (SC)	Week 6	OR 0.09 (95% CI 0.01–0.65); p = 0.018		
	BWT (SC) (threshold ≤2.7 mm)	Week 8-26	AUROC 0.95 (95% CI 0.88–1.00, p<0.0001)<br>Sensitivity: 100% (95% CI, 60%-100%) Specificity: 86% (95% CI, 64%-96%). PPV: 73%. NPV: 1		
	BWT (DC) (threshold ≤3.62 mm)	Week 2	AUROC 0.87 (95% CI 0.71–1.00, p=0.006)		
	BWT (DC) (threshold ≤2.62 mm)	Week 8-26	AUROC 0.96 (95% CI 0.87–1.00, p=0.001)		
Alloca, 2023	MUC ≤ 6.2	Week 12	OR 10.41, 95% CI 1.09– 99.29; p=0.041	MES = 0	

	MUC (≥2 reduction)	Baseline vs. week 12	AUROC 0.82, 95% CI 0.68–0.91		Mean 9.4 ± 3.6 months
	MUC ≤ 4.3	Week 12	AUROC 0.88, 95% CI 0.75–0.95		
Endoscopic response / improvement					
De Voogd 2021	HoS	Baseline (prior to starting treatment)	Obs lack of endoscopic improvement: OR 0.16 (95% CI: 0.04–0.65, p=0.008)	EMS≤1	8-26 weeks
De Voogd 2023	CDS (SC) per category increase	Week 6	OR 0.35 (95% CI 0.14–0.88); p=0.026	EMS ≤1 (endoscopic improvement)	8-26 weeks
	Submucosal thickness, pr mm increase (SC)	Week 6	OR 0.14 (95% CI 0.03–0.75); p=0.020.		
	Return of haustrations	Week 6	OR 13.50 (95% CI 2.01–90.69); p=0.007	EMS decrease ≥1 (endoscopic response)	
	BWT (SC) (threshold ≤3.5)	Week 8-26	AUROC 0.96 (95% CI 0.88-1.00 p<0.0001) Sensitivity: 92% (95% CI, 64%- 100%) Specificity: 87% (95% CI, 60%-98%). PPV: 87%. NPV: 93%	EMS ≤1 (endoscopic improvement)	
	BWT relative reduction ≥23%	Week 8-26	AUROC, 0.81; 95% CI, 0.61-1.00; p=0.019) Sensitivity: 77% (95% CI, 46%-94%). Specificity: 81% (95% CI, 0.54%-0.95%). PPV: 77%. NPV: 81%	EMS ≥1 (endoscopic response)	
Alloca, 2023	MUC ≤ 6.2	Week 12	[OR] 5.80, 95% CI 1.49–22.47; p=0.010	Endoscopic improvement MES ≤ 1 after	Mean 9.4 ± 3.6 months
	MUC (≥2 reduction)	Baseline vs. week 12	AUROC 0.806, 95% CI 0.667–0.904		
Pruijt 2025	RSE (SC)	Baseline	OR 0.98 (95% CI 0.97-0.99), p=0.007 AUROC (0.76 95% CI 0.61-0.92), p=0.003)	Endoscopic response: ≥1 point decrease in EMS	9.6 (8.7-20.4) weeks
	RSE > 108 greyscale units		OR 0.07 (95% CI 0.01–0.45)		

Peng 2024	BWT >28% decrease	Baseline vs. post-induction (not specified)	AUROC 0.743; p=0.015	Decrease in MES ≥1 or a reduction in UCEIS ≥2	Treatment induction period (duration not specified)
	Normal BWS	Baseline	[OR] 23.334, 95% CI 2.257-241.219; p=0.008		
	BWT, CDS, presence of lymph nodes, i-fat,		Not independent predictors of endoscopic response (on multivariable analysis)		
**Colectomy**					
Sed 2024	MUC	Baseline	AUROC 0.83 (95% CI: 0.75–0.92)	Colectomy during follow-up	1.8 [1.0–2.7] years
	MUC threshold ≥ 7.7mm		Sensitivity: 1.0. Specificity: 0.6, PLR: of 2.5. NLR: 0		
	BWT		AUROC 0.80 (95% CI: 0.71–0.90)		
	BWT threshold ≥ 4.6mm		Optimal cut-off value as assessed by the Youden index Sensitivity: 0.85 Specificity: 0.60, PLR: 2.3. NLR:0.3		
	BWF presence		AUROC 0.77 (95% CI: 0.73–0.82)		
Madsen 2025	BWT	Baseline	OR 2.5 (95%CI: 1.7-4.1, p<0.001) Multivariable analysis: OR 2.0 (95% CI: 1.2-3.3, p<0.1) The optimal thresholds were BWT >6 mm (AUC: 0.85, sensitivity: 80%, specificity: 90%)	Colectomy during follow-up	3 months
	CDS		OR 4.9 (95% CI: 2.0-2.6, p<0.01)		
	Loss of BWS		OR 3.4 (95% CI: 1.8-7.9, p<0.001)		
	I-fat		OR 3.6 (95% CI: 1.7-9.5, p<0.01)		
	IBUS-SAS		Optimal threshold IBUS- SAS >42 (AUC: 0.88, sensitivity: 100%, specificity: 62%)		
**Treatment failure**					
De Voogd 2022	HoS	Baseline (prior to starting treatment)	OR 4.44 (95% CI: 1.08–18.32, p=0.03)	Failure of 1 biologic	8-26 weeks
			OR 5.63 (95% CI: 1.54–20.52, p=0.009)	Failure of >1 biologics	

Alloca 2022	MUC > 6.2	Baseline	HR: 3.87, 95% CI: 2.25–6.64, p < 0.001	UC-related negative course: need for corticosteroids, treatment escalation, hospitalization, or need for colectomy	1.6 (0.9-2.7) years
Madsen 2025	Transmural remission (no inflammation present, evaluated by CDS, BWT, BWS, I-fat)	3 months	Patients in transmural remission: 6%<br>Patients not in transmural remission: 19%<br>p=0.04	Requiring systemic steroids	1 year
Clinical remission					
Zorzi 2021	Lower BWT	Baseline	OR 0.19, 95% CI 0.05-0.72, p=0.0023	pMayo Score = 0-1	3 months
	MUC	Baseline	OR 0.48, 95% CI 0.21-0.83, p=0.001		
Madsen 2025	Transmural remission (no inflammation present, evaluated by CDS, BWT, BWS, I-fat)	3 months	Among patients NOT in transmural remission: 34% in steroid free clinical remission<br>Among patients in transmural remission: 87% in steroid free clinical remission<br>p<0.001	Steroid free clinical remission at 6 months	6 months
			Among patients NOT in transmural remission: 59% in steroid free clinical remission<br>Among patients in transmural remission: 82% in steroid free clinical remission<br>p=0.031	Steroid free clinical remission at 9 months	9 months

			Among patients NOT in transmural remission: 59% in steroid free clinical remission<br>Among patients in transmural remission: 82% in steroid free clinical remission<br>p=0.031	Steroid free clinical remission at 12 months	12 months
Clinical respons					
Goeretz 2018	WiR	Baseline vs. week 14	Response vs. non-response p=0.037	Clinical mayo subscore Δ ≥ -1	Week 14
Helwig, 2025	BWT reduction ≥ 25%	Baseline vs. week 6	OR = 13.91, 95% CI 1.13–1986.85	Vedolizumab ≥ 24 μ g/ml, ≥ 25% reduction in colon wall thickness, or ≥ 50% reduction in feacal calprotectin	Week 14-22

Evidence table for data concerning Ulcerative Colitis Outpatients. **Colonic Segments**: **SC**: Sigmoid Colon, **DC**: Descending Colon**. Endoscopic Indices: MES**: Mayo Endoscopic Subscore**, EMS**: Endoscopic Mayo Score**, UCEIS**: Ulcerative Colitis Endoscopic Index of Severity. **Clinical Scores: pMayo score**: Partial Mayo Score**. Ultrasound Parameters, BWS**: Bowel Wall Stratification**, WLS**: Wall Layer Stratification**, BWT** Bowel Wall Thickness, **BWF:** Bowel Wall Flow. **CDS**: Color Doppler Signal**, HoS**: Hyperechogenicity of the Submucosa**, RSE**: Relative Submucosal Echogenicity**, WiR**: Wall Inflammation Ratio**. Composite Ultrasound Scores: MUC**: Milan Ultrasound Criteria (MUC = 1.4 × BWT + 2 × CDS). **IBUS-SAS:** International Bowel Ultrasound Segmental Activity Score.

Supplementary Table S7: PRISMA Checklist.

Section and Topic	Item #	Checklist item	Location where item is reported
**TITLE**			
Title	1	Identify the report as a systematic review.	Page 1
**ABSTRACT**			
Abstract	2	See the PRISMA 2020 for Abstracts checklist.	Page 3
**INTRODUCTION**			
Rationale	3	Describe the rationale for the review in the context of existing knowledge.	Page 5-6
Objectives	4	Provide an explicit statement of the objective(s) or question(s) the review addresses.	Page 6
**METHODS**			
Eligibility criteria	5	Specify the inclusion and exclusion criteria for the review and how studies were grouped for the syntheses.	Page 7
Information sources	6	Specify all databases, registers, websites, organizations, reference lists, and other sources searched or consulted to identify studies. Specify the date when each source was last searched or consulted.	Page 7 (no date provided)
Search strategy	7	Present the full search strategies for all databases, registers and websites, including any filters and limits used.	Supplementary table 1
Selection process	8	Specify the methods used to decide whether a study met the inclusion criteria of the review, including how many reviewers screened each record and each report retrieved, whether they worked independently, and if applicable, details of automation tools used in the process.	Page 7
Data collection process	9	Specify the methods used to collect data from reports, including how many reviewers collected data from each report, whether they worked independently, any processes for obtaining or confirming data from study investigators, and if applicable, details of automation tools used in the process.	Page 7
Data items	10a	List and define all outcomes for which data were sought. Specify whether all results that were compatible with each outcome domain in each study were sought (e.g. for all measures, time points, analyses), and if not, the methods used to decide which results to collect.	Page 7
	10b	and define all other variables for which data were sought (e.g. participant and intervention characteristics, funding sources). Describe any assumptions made about any missing or unclear information.	Page 7

Section and Topic	Item #	Checklist item	Location where item is reported
Study risk of bias assessment	11	the methods used to assess risk of bias in the included studies, including details of the tool(s) used, how many reviewers assessed each study and whether they worked independently, and if applicable, details of automation tools used in the process.	Page 8 + supplementary table 2
Effect measures	12	Specify for each outcome the effect measure(s) (e.g. risk ratio, mean difference) used in the synthesis or presentation of results.	Not applicable
Synthesis methods	13a	Describe the processes used to decide which studies were eligible for each synthesis (e.g. tabulating the study intervention characteristics and comparing against the planned groups for each synthesis (item #5)).	Not applicable, as no data synthesis was performed
	13b	Describe any methods required to prepare the data for presentation or synthesis, such as handling of missing summary statistics, or data conversions.	Not applicable, as no data synthesis was performed
	13c	Describe any methods used to tabulate or visually display results of individual studies and syntheses.	Not applicable, as no data synthesis was performed
	13d	Describe any methods used to synthesize results and provide a rationale for the choice(s). If meta-analysis was performed, describe the model(s), method(s) to identify the presence and extent of statistical heterogeneity, and software package(s) used.	Not applicable, as no data synthesis was performed
	13e	Describe any methods used to explore possible causes of heterogeneity among study results (e.g. subgroup analysis, meta-regression).	Not applicable, as no data synthesis was performed
	13f	Describe any sensitivity analyses conducted to assess robustness of the synthesized results.	Not applicable, as no data

Section and Topic	Item #	Checklist item	Location where item is reported
			synthesis was performed
Reporting bias assessment	14	Describe any methods used to assess risk of bias due to missing results in a synthesis (arising from reporting biases).	Not applicable, as no data synthesis was performed
Certainty assessment	15	Describe any methods used to assess certainty (or confidence) in the body of evidence for an outcome.	Not applicable, as no data synthesis was performed
**RESULTS**			
Study selection	16a	Describe the results of the search and selection process, from the number of records identified in the search to the number of studies included in the review, ideally using a flow diagram.	Figure 1
	16b	Cite studies that might appear to meet the inclusion criteria, but which were excluded, and explain why they were excluded.	Not provided
Study characteristics	17	Cite each included study and present its characteristics.	Supplementary table 4
Risk of bias in studies	18	Present assessments of risk of bias for each included study.	Supplementary table 3
Results of individual studies	19	For all outcomes, present, for each study: (a) summary statistics for each group (where appropriate) and (b) an effect estimate and its precision (e.g. confidence/credible interval), ideally using structured tables or plots.	Supplementary table 5 + supplementary table 6
Results of syntheses	20a	For each synthesis, briefly summarize the characteristics and risk of bias among contributing studies.	Not applicable, as no data synthesis was performed

Section and Topic	Item #	Checklist item	Location where item is reported
	20b	Present results of all statistical syntheses conducted. If meta-analysis was done, present for each the summary estimate and its precision (e.g. confidence/credible interval) and measures of statistical heterogeneity. If comparing groups, describe the direction of the effect.	Not applicable, as no data synthesis was performed
	20c	Present results of all investigations of possible causes of heterogeneity among study results.	Not applicable, as no data synthesis was performed
	20d	Present results of all sensitivity analyses conducted to assess the robustness of the synthesized results.	Not applicable, as no data synthesis was performed
Reporting biases	21	Present assessments of risk of bias due to missing results (arising from reporting biases) for each synthesis assessed.	Not applicable, as no data synthesis was performed
Certainty of evidence	22	Present assessments of certainty (or confidence) in the body of evidence for each outcome assessed.	Not applicable, as no data synthesis was performed
**DISCUSSION**			
Discussion	23a	Provide a general interpretation of the results in the context of other evidence.	Page 14, 15, 16
	23b	Discuss any limitations of the evidence included in the review.	Page 16
	23c	Discuss any limitations of the review processes used.	Page 16
	23d	Discuss implications of the results for practice, policy, and future research.	Page 14, 15, 16, 17
**OTHER INFORMATION**			

**Section and Topic**	**Item #**	**Checklist item**	**Location where item is reported**
Registration and protocol	24a	Provide registration information for the review, including register name and registration number, or state that the review was not registered.	Page 7
	24b	Indicate where the review protocol can be accessed, or state that a protocol was not prepared.	Page 7
	24c	Describe and explain any amendments to information provided at registration or in the protocol.	Not applicable
Support	25	Describe sources of financial or non-financial support for the review, and the role of the funders or sponsors in the review.	Page 1, 2
Competing interests	26	Declare any competing interests of review authors.	Page 1, 2
Availability of data, code and other materials	27	Report which of the following are publicly available and where they can be found: template data collection forms; data extracted from included studies; data used for all analyses; analytic code; any other materials used in the review.	Everything that is publicly available is included in the review document

From: Page MJ, McKenzie JE, Bossuyt PM, Boutron I, Hoffmann TC, Mulrow CD, et al. The PRISMA 2020 statement: an updated guideline for reporting systematic reviews. BMJ 2021;372:n71. doi: 10.1136/bmj.n71. This work is licensed under CC BY 4.0. To view a copy of this license, visit https://creativecommons.org/licenses/by/4.0/
